# Antimicrobial Resistance in the Environment: Towards Elucidating the Roles of Bioaerosols in Transmission and Detection of Antibacterial Resistance Genes

**DOI:** 10.3390/antibiotics11070974

**Published:** 2022-07-19

**Authors:** Paul B. L. George, Florent Rossi, Magali-Wen St-Germain, Pierre Amato, Thierry Badard, Michel G. Bergeron, Maurice Boissinot, Steve J. Charette, Brenda L. Coleman, Jacques Corbeil, Alexander I. Culley, Marie-Lou Gaucher, Matthieu Girard, Stéphane Godbout, Shelley P. Kirychuk, André Marette, Allison McGeer, Patrick T. O’Shaughnessy, E. Jane Parmley, Serge Simard, Richard J. Reid-Smith, Edward Topp, Luc Trudel, Maosheng Yao, Patrick Brassard, Anne-Marie Delort, Araceli D. Larios, Valérie Létourneau, Valérie E. Paquet, Marie-Hélène Pedneau, Émilie Pic, Brooke Thompson, Marc Veillette, Mary Thaler, Ilaria Scapino, Maria Lebeuf, Mahsa Baghdadi, Alejandra Castillo Toro, Amélia Bélanger Cayouette, Marie-Julie Dubois, Alicia F. Durocher, Sarah B. Girard, Andrea Katherín Carranza Diaz, Asmaâ Khalloufi, Samantha Leclerc, Joanie Lemieux, Manuel Pérez Maldonado, Geneviève Pilon, Colleen P. Murphy, Charly A. Notling, Daniel Ofori-Darko, Juliette Provencher, Annabelle Richer-Fortin, Nathalie Turgeon, Caroline Duchaine

**Affiliations:** 1 Département de Médecine Moléculaire, Université Laval, Quebec City, QC G1V 0A6, Canada; paul.george@bcm.ulaval.ca (P.B.L.G.); jacques.corbeil@fmed.ulaval.ca (J.C.); ilaria.scapino.1@ulaval.ca (I.S.); 2 Département de Biochimie, de Microbiologie et de Bio-Informatique, Université Laval, Quebec City, QC G1V 0A6, Canada; florent.rossi@uca.fr (F.R.); magali-wen.st-germain.1@ulaval.ca (M.-W.S.-G.); steve.charette@bcm.ulaval.ca (S.J.C.); alexander.culley@bcm.ulaval.ca (A.I.C.); luc.trudel@bcm.ulaval.ca (L.T.); valerie.paquet.2@ulaval.ca (V.E.P.); mary.thaler.1@ulaval.ca (M.T.); mahsa.baghdadi.1@ulaval.ca (M.B.); amelia.belanger-cayouette.1@ulaval.ca (A.B.C.); alicia.durocher.1@ulaval.ca (A.F.D.); sarah.girard.7@ulaval.ca (S.B.G.); asmaa.khalloufi@umontreal.ca (A.K.); samantha.leclerc.3@ulaval.ca (S.L.); joanie.lemieux.2@ulaval.ca (J.L.); juliette.provencher.1@ulaval.ca (J.P.); annabelle.richer-fortin.1@ulaval.ca (A.R.-F.); 3 Institut de Chimie de Clermont-Ferrand, SIGMA Clermont, CNRS, Université Clermont-Auvergne, 63178 Clermont-Ferrand, France; pierre.amato@uca.fr (P.A.); a-marie.delort@uca.fr (A.-M.D.); 4 Centre de Recherche de L’Institut Universitaire de Cardiologie et de Pneumologie de Québec, Quebec City, QC G1V 4G5, Canada; andre.marette@criucpq.ulaval.ca (A.M.); serge.simard@criucpq.ulaval.ca (S.S.); valerie.letourneau@criucpq.ulaval.ca (V.L.); marie-helene.pedneau@mat.ulaval.ca (M.-H.P.); marc.veillette@criucpq.ulaval.ca (M.V.); maria.lebeuf.1@ulaval.ca (M.L.); marie-julie.dubois@criucpq.ulaval.ca (M.-J.D.); genevieve.pilon@criucpq.ulaval.ca (G.P.); nathalie.turgeon@criucpq.ulaval.ca (N.T.); 5 Centre de Recherche en Données et Intelligence Géospatiales (CRDIG), Quebec City, QC G1V 0A6, Canada; thierry.badard@scg.ulaval.ca; 6 Centre de Recherche en Infectiologie, Centre de Recherche du CHU de Québec-Université Laval, Axe Maladies Infectieuses et Immunitaires, Quebec City, QC G1V 4G2, Canada; michel.g.bergeron@crchudequebec.ulaval.ca (M.G.B.); maurice.boissinot@crchudequebec.ulaval.ca (M.B.); emilie.pic@crchudequebec.ulaval.ca (É.P.); 7 Institut de Biologie Intégrative et des Systèmes, Université Laval, Quebec City, QC G1V 0A6, Canada; 8 Dalla Lana School of Public Health, University of Toronto, Toronto, ON M5T 3M7, Canada; brenda.coleman@sinaihealth.ca (B.L.C.); allison.mcgeer@sinaihealth.ca (A.M.); 9 Research Chair in Meat Safety, Département de Pathologie et Microbiologie, Université de Montréal, Saint-Hyacinthe, QC J2S 2M2, Canada; marie-lou.gaucher@umontreal.ca; 10 Agrivitia Canada, Saskatoon, SK S7N 2Z4, Canada; matthieu.girard@usask.ca; 11 Institut de Recherche et de Développement en Agroenvironnement (IRDA), Quebec City, QC G1P 3W8, Canada; stephane.godbout@irda.qc.ca (S.G.); dalila.larios@irda.qc.ca (A.D.L.); andrea-katherin.carranza-diaz.1@ulaval.ca (A.K.C.D.); 12 Département des Sols et de Génie Agroalimentaire, Université Laval, Quebec City, QC G1V 0A6, Canada; patrick.brassard@irda.qc.ca; 13 Department of Medicine, University of Saskatchewan, Saskatoon, SK S7N 0X8, Canada; shelley.kirychuk@usask.ca (S.P.K.); brooke.thompson@usask.ca (B.T.); alejandracastillo@javeriana.edu.co (A.C.T.); can291@mail.usask.ca (C.A.N.); 14 Institut sur la Nutrition et les Aliments Fonctionnels, Université Laval, Quebec City, QC G1V 0A6, Canada; 15 Department of Laboratory Medicine and Pathobiology, University of Toronto, Toronto, ON M5S 1A8, Canada; 16 Department of Occupational and Environmental Health, The University of Iowa, Iowa City, IA 52246, USA; patrick-oshaughnessy@uiowa.edu; 17 Canadian Wildlife Health Cooperative, University of Guelph, Guelph, ON N1G 2W1, Canada; jparmley@uoguelph.ca; 18 Department of Population Medicine, University of Guelph, Guelph, ON N1G 2W1, Canada; richard.reid-smith@phac-aspc.gc.ca (R.J.R.-S.); mperezma@uoguelph.ca (M.P.M.); 19 Centre for Foodborne, Environmental and Zoonotic Infectious Diseases, Public Health Agency of Canada, Guelph, ON N1G 3W4, Canada; colleen.murphy@phac-aspc.gc.ca (C.P.M.); daniel.ofori-darko@phac-aspc.gc.ca (D.O.-D.); 20 Agriculture and Agri-Food Canada, London Research and Development Centre, London, ON N5V 4T3, Canada; ed.topp@agr.gc.ca; 21 Department of Biology, The University of Western Ontario, London, ON N6A 5B7, Canada; 22 State Key Joint Laboratory of Environmental Simulation and Pollution Control, College of Environmental Sciences and Engineering, Peking University, Beijing 100871, China; yao@pku.edu.cn; 23 Tecnológico Nacional de México/ITS de Perote, Perote 91270, Mexico

**Keywords:** antibiotic resistance genes, large-scale monitoring, one Health, culturomics, DNA sequencing, quantitative PCR, bioaerosols

## Abstract

Antimicrobial resistance (AMR) is continuing to grow across the world. Though often thought of as a mostly public health issue, AMR is also a major agricultural and environmental problem. As such, many researchers refer to it as the preeminent One Health issue. Aerial transport of antimicrobial-resistant bacteria via bioaerosols is still poorly understood. Recent work has highlighted the presence of antibiotic resistance genes in bioaerosols. Emissions of AMR bacteria and genes have been detected from various sources, including wastewater treatment plants, hospitals, and agricultural practices; however, their impacts on the broader environment are poorly understood. Contextualizing the roles of bioaerosols in the dissemination of AMR necessitates a multidisciplinary approach. Environmental factors, industrial and medical practices, as well as ecological principles influence the aerial dissemination of resistant bacteria. This article introduces an ongoing project assessing the presence and fate of AMR in bioaerosols across Canada. Its various sub-studies include the assessment of the emissions of antibiotic resistance genes from many agricultural practices, their long-distance transport, new integrative methods of assessment, and the creation of dissemination models over short and long distances. Results from sub-studies are beginning to be published. Consequently, this paper explains the background behind the development of the various sub-studies and highlight their shared aspects.

## 1. Introduction

Rising levels of antimicrobial resistance (AMR) have caused great concern amongst policymakers, doctors, and governments in recent years. In 2019, an estimated 4.95 million deaths were associated with AMR globally, of which approximately 1.27 million were directly attributable to resistant bacteria [1]. These data likewise revealed stark geographic trends, with the greatest number of deaths due to AMR found in sub-Saharan Africa and south Asia, whereas the fewest were observed in Australasia [1]. Unfortunately, this number is expected to increase, and consequently, so will healthcare and economic costs. In Canada, AMR could reduce GDP projections by 13–21 billion CAD by 2050, with an increase in associated healthcare costs from 1.4 billion CAD in 2018 to 8 billion CAD in 2050 [2]. The knock-on effects of the SARS-CoV2 pandemic exemplify the extent to which novel or re-emerging pathogens pressure economic [3] and healthcare systems [4]. Yet the problem of AMR represents a convergence of many factors, such as antimicrobial overuse and misuse, pollution [5], and natural coevolution dynamics [6], and requires a multidisciplinary approach to mitigate adverse outcomes.

This reality has led scientists and policymakers to consider AMR as the preeminent One Health question [7]. Much attention has been directed to studying the spread of AMR—particularly antimicrobial resistance genes (ARGs)—in the environment reviewed in [5]. To date, most work focused on ARGs in the broader environment has been conducted in soils, water bodies, or wildlife [5,8]. For example, animal production is the leading consumer of antimicrobial agents globally [9], accounting for ~78% of agents used in Canada in 2018 [10]. Run-off from manure application or livestock barns introduces unprocessed antimicrobial compounds and resistant organisms to soils and watercourses, allowing ARGs to spread to populations of naïve microbes via horizontal gene transfer [5]. However, airborne ARG dispersal is increasingly recognized as an important, though severely understudied, route for disseminating AMR in the environment [5,11,12].

Indeed, Huijbers and collaborators [8] reported 157 studies of AMR bacteria in the environment as of 2014. Only 5 of these studies (3%) looked at bioaerosols or settled dust, compared to 25 for soil, 56 for water, and 71 for wildlife. In the intervening years, there has been a marked increase in the number of studies assessing airborne ARGs in the environment. Notably, bioaerosols are known to influence the dispersal of resistant microorganisms through wildlife, domestic animals, soil, water, and humans [8]. Studies have reported ARG-laden bioaerosols detected approximately 2 km from agricultural buildings [13] and projected dispersal footprints of up to 10 km [14]. This is of great concern as bioaerosol emissions are challenging to control.

Understanding the role bioaerosols play in ARG transmission is critical to addressing AMR in the environment. Yet this requires a multidisciplinary approach that can integrate data from diverse systems, including indoor (i.e., wastewater treatment, livestock buildings) and outdoor environments (i.e., agricultural fields, urban air) or remote areas (i.e., clouds, Canadian North, overseas) and produce data that can be integrated into the current understanding of AMR. To this end, we have launched a multi-year research program dedicated to the airborne dissemination of ARGs. The project is funded by a Natural Sciences and Engineering Research Council of Canada (NSERC) Discovery Frontiers opportunity, specifically targeting AMR in the environment (2019 competition), as a major research topic of the Canada Research Chair on Bioaerosols and a network of Canadian and international collaborators. Here, we outline the rationale for this undertaking and describe the ongoing research topics. The purpose of this article is to present (i) the current knowledge gaps in the roles of bioaerosols in AMR; (ii) our ongoing work to address them; (iii) stimulating discussion and interest amongst the broader scientific community to further shed light on the roles of bioaerosols in disseminating ARGs; and (iv) open doors for new collaborations.

## 2. Bioaerosols

Bioaerosols are particles suspended in the air upon which, microorganisms, living or dead, microbial fragments, and viruses may be found. Such biological material can be transported over many kilometres in the environment. Bioaerosols are defined as particles of less than 100 μm and may remain airborne indefinitely, depending on air currents and turbulence [15]. The aerial microbial community has been studied for over 150 years, notably heralded by Pasteur’s observations of airborne bacteria [16]. Sources of bioaerosol emissions are now well known and include natural and stochastic events (wind, raindrops) and anthropogenic-associated ones, such as wastewater treatment and agricultural practices [17]. Much of the literature on bioaerosols has focused on disease propagation [18,19] and food production processes like brewing [20]. Increasingly, occupational, industrial, and agricultural bioaerosols have been studied, providing a better understanding of their ecology and biodiversity. However, a recent synthesis by Šantl-Temkiv and colleagues highlighted significant gaps in our understanding of outdoor bioaerosols [21]. In particular, they highlighted an absence of knowledge surrounding airborne communities in natural and built environments, their emission rates in natural environments, and the impacts of anthropogenic change on airborne microorganisms. Addressing these knowledge gaps requires new and integrative methodological approaches.

Bioaerosols are representative of their sources and together form a combined sample of multiple origins, although a recent hypothesis tends to support the existence of a specific atmospheric microbiota [22]. Following transport by bioaerosols, microorganisms can colonize depositional environments, cause infections, or simply decay after settling; additionally, their genetic material (free or within cells) can be transferred throughout the environment. Despite the heavy research focus on human-associated bioaerosols, most bioaerosols are derived from plants, soil [23,24], and natural bodies of water [25]. Increasingly, researchers have attempted to track the fate of bioaerosols from anthropogenic sources [11,13,14].

Often researchers rely on modeling approaches or database references to infer the source community of bioaerosols [26]. Since viable microorganisms can travel thousands of kilometres by air [27], the role of bioaerosols as a vector for the dissemination of ARGs is an increasingly important area of research. These organisms may exhibit AMR, making their aerial transport a significant health concern. Many studies have detected potentially pathogenic taxa from the air, such as *Legionella* [28,29] and *Staphylococcus* [14,30] using DNA-based methods and culturomics—the use of multiple media and growth conditions to better facilitate the isolation of bacteria under controlled conditions. 

Bioaerosols may play an active role in the dissemination of ARGs in the environment. In Colorado, concentrated animal feeding operations were shown to emit ARGs detectable over 2 km from buildings [13]. Furthermore, non-agricultural indoor environments such as clinics and homeless shelters were found to be a source of ARG in the broader environment [13]. The richness of ARG types in urban smog exposed to pharmaceutical pollution is higher than in wastewater or sludge [31]. The high taxonomic and genetic bacterial diversity of outdoor environments indicates that bioaerosols are a vast reservoir of ARGs with the potential to be transferred to pathogenic agents.

Particulate matter (PM) present in pollution events harbours ARGs in greater concentrations than under ambient conditions [31]. Several studies have revealed the presence of ARGs in PM in urban areas [31,32,33] of various cities and the air is now a suspected transmission route for AMR bacteria from point sources such as wastewater treatment plants. A recent study assessed the distribution of ARGs worldwide using automobile cabin filters and found marked geographical variations [33]. Additionally, laboratory studies have shown that compounds found in vehicle exhaust stimulate bacterial stress responses, including promoting the expression of plasmid transfer genes, potentially accelerating ARG transfer in urban air [34].

Recent research has shown that ARGs are present in the indoor air of wastewater treatment facilities [35] and livestock buildings [14,36,37]. It is expected that ventilation will expel high concentrations of ARG-laden bioaerosols into the environment. Other activities, such as manure application, generate bioaerosols that may contain medically important bacteria or functional genes over wide areas [38]. It is unknown whether occupational exposure to these organisms in outdoor environments poses health risks. ARG transfer from the environment to humans is poorly understood, though limited evidence has found that specific pathways, mainly via water, are viable [39]. Further work is needed to integrate ambient bioaerosols into exposure models.

Nevertheless, the above examples provide evidence that a significant number of ARGs are present in bioaerosols. For instance, in their worldwide sampling of dust collected from vehicle air filters, Li et al. detected ARGs against aminoglycosides, beta-lactams, macrolides, quinolones, sulfonamides, tetracyclines, and vancomycin [33]. Bioaerosols containing tetracycline ARGs have been observed in many different locations worldwide [33]; specific genes such as *tetM* and *tetO* have been found in the air of agricultural buildings and farms [14,37]. Furthermore, in healthcare settings, the *tetW* gene was common in health clinics and a homeless shelter in Colorado, USA [13]. In a South Carolina, USA wastewater treatment plants, macrolide resistance genes *ermB* and *ermC* were highly abundant [35]. Yet, there is still much work to be carried out to identify ARGs in bioaerosols and link them to source locations.

## 3. The Frontiers Project

### 3.1. Project Members

**Figure 1:**
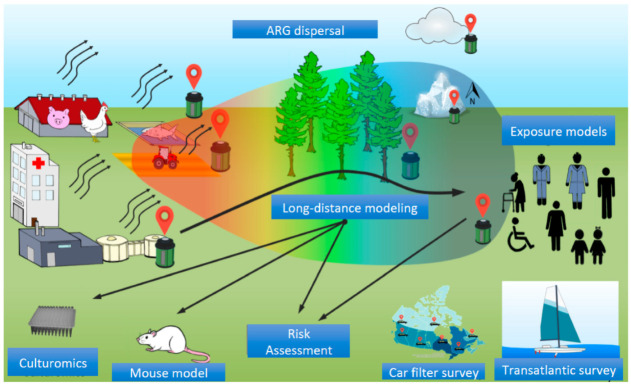
Graphical summary of the research program. Depicted are representations of the environments and objectives of the overarching project. Samples from human-associated sampling environments include livestock buildings, fish farms, arable fields, hospitals, and wastewater treatment plants to investigate ARG emissions. Environmental samples will also be taken from clouds, the Canadian North, a transatlantic survey, vehicle filters collected from sites across Canada, and conifer needles to inform the long-distance dispersal of ARGs. The data generated in these projects will inform culturomics and enrichment experiments, in vivo ARG transfer studies in animal models, and exposure models in humans. Finally, selected data will be used to inform risk assessment models.

Dr. Caroline Duchaine has spearheaded a multi-year project funded by an NSERC Discovery Frontiers program to study ARGs in bioaerosols to address these fundamental questions. The overarching project incorporates studies of indoor environments, their emissions into the environment and their long-distance transport, novel ARG tracking and surveillance methods, selective culture approaches, and animal models that can be incorporated into exposure and risk assessment models (Figure 1). The aspects of each sub-study can be categorized into several topical areas but at this stage can be best summarized by methodological approaches and study environments. All aspects of the project follow a One Health approach. Table 1 presents a summary of all types of samples collected, sampling sites, number of samples, and expected outcomes.

This project is led by the Canada Research Chair in Bioaerosols based in the bioaerosol laboratory, *Institut Universitaire de Cardiologie et Pneumologie de Québec*, Université Laval in Quebec City, QC, Canada. A multidisciplinary team of collaborators at Université Laval, across Canada (University of Saskatchewan, Université de Montréal, University of Guelph, Western University, Public Health Agency of Canada, Agriculture and Agri-Food Canada, Mount Sinai Research Institute) and internationally (Université Clermont Auvergne, France; University of Iowa, IA, USA; Peking University, China) are participating in the project. They bring expertise in human, veterinary and aquacultural disease, antibiotics, virology, bioaerosols, culture approaches, geography, modeling, bioinformatics, and artificial intelligence.

Together, our team proposes to estimate the contribution of agricultural and sanitation activities to ARG dispersal across Canada and the potential for long-distance transfers through a program addressing the following objectives: (i) assess ARG dispersion and associated bacterial diversity across Canada and their relationships to land use via vehicle cabin filters; (ii) assess ARG dispersion and associated bacterial diversity of bioaerosols in representative source locations by using high-volume air samplers; (iii) determine the subsequent fate of airborne ARGs; (iv) determine the potential for long-distance transport of ARGs; and (v) adding the role of bioaerosols to an integrated assessment model on AMR. The findings will significantly enhance the understanding of ARGs in bioaerosols and provide a framework for future research. It is hoped that the project will stimulate other researchers to pursue interdisciplinary approaches to studying bioaerosols.

### 3.2. Bioaerosol Sampling

#### 3.2.1. Short Distance Air Sampling and Local Emission Sources Determination

Evaluating the contributions of various bioaerosol sources is key to exposure and mitigation strategies. Hospitals, wastewater treatment plants, fish farms, livestock barns —particularly those of swine and poultry—and manure spreading are being evaluated in this research. Assessing the emissions of bioaerosols within and around these locations requires the use of multiple sampling methodologies involving a range of air samplers (Table 2). These instruments physically collect air and concentrate PM either through filtration or via gravitational forces reviewed in [40]. Air samples will be collected at multiple locations: inside buildings and both up- and downwind of emission sources. Active high-volume air sampling will be performed using a wide range of air samplers (Table 2) to evaluate bacterial diversity and ARG profile up- and downwind from source locations. Upwind samples are collected to establish an ambient profile of airborne organisms to which inputs from source locations can be compared.

While frequently used in indoor environments, the deployment of such samplers outdoors presents challenges. Decisions in deployment location (both on the ground and at height) and duration may introduce sampling biases into collection. Sampling is also limited to relatively short periods, which may not align with ideal weather conditions for bioaerosol collection. Standardized sampling approaches have been implemented by all investigators to reduce bias throughout the project. The SASS3100 (Research International, Seattle, WA, USA) electret sampler is part of most of the sampling campaigns. However, it is unsuitable for culture techniques and does not maintain microbial viability. For culture and viability purposes, liquid samplers will be added to protocols, such as the SASS2300 and Coriolis μ (Bertin-Instruments, Montigny-le-Bretonneux, France), where appropriate.

In terms of study locations, these sampling campaigns will be undertaken in several locations across the Canadian provinces of Quebec, Ontario, and Saskatchewan. Several wastewater treatment plants have been selected for study. The presence of ARGs in bioaerosols was shown by previous work from our research team in swine barns [36,41] and poultry barns [42,43] in Quebec and Saskatchewan. However, the emissions of ARGs from these buildings, their subsequent local dispersion and their contribution to the long-range exposure are not well documented. To that end, comparisons will be drawn from farms in these two provinces, where intensive agriculture is common, as well as between conventional and antibiotic-free farms. Impacts of various manure spreading techniques on bioaerosol formation and composition are underway at the *Institut de Recherche et de Développement en Agroenvironnement* (IRDA) farm at Saint-Lambert-de-Lauzon, Quebec. Several Quebec fish farms, including the *Laboratoire Aquatique de Recherche enSsciences Environnementales et Médicales* (LARSEM) will also be studied. Air will be sampled in hospitals in Toronto, ON, Canada. 

#### 3.2.2. Long-Distance ARG Transport

Assessing the long-distance transport of ARGs is a critical objective of this project. We will address this question using a variety of complementary approaches. The aerial microbiome of remote locations in the Canadian North will be characterized. Arctic bioaerosols are a critical blind spot in our understanding of airborne biodiversity [21], which experience a pronounced set of environmental pressures from a warming climate and changing population demographics. Losses of polar ice and permafrost may increase aerosolization rates of particulate matter [44] and, thereby augment the polar bioaerosol community. Bioaerosols of pristine sites (Ward Hunt Island, Nunavut, Canada) and an Inuit community (Resolute Bay, Nunavut, Canada) will be collected. Bioaerosols are expected to be very diluted in these areas, so a complementary approach using a large volume concentrator (SASS4100) and small volume extractors (i.e., SASS3100) will be used to maximize sampling potential.

The resistome of clouds will also be characterized. Clouds can be considered as an oases for microorganisms, providing them with more favourable conditions such as water or shading against UV radiation [22]. Such situations can potentially affect their atmospheric transport and, therefore, facilitate the atmospheric dispersion of ARGs worldwide. This project will undertake cloud samplings at the Puy-de-Dôme meteorological station in Clermont-Ferrand, France (1465 m elevation). Here the continuous collection of multiple physical and meteorological parameters is undertaken by a team of atmospheric microbiologists. Bacterial community and ARG content in cloud water will be assessed and related to the geographical origin of air masses and their physical and chemical features. Clouds have been sampled using cloud droplet impactors [45] to allow for the characterization of cloud water chemical properties. In parallel, recently developed high flow rate impingers filled with a nucleic acid preservative solution will be used [46] for the molecular monitoring of ARGs.

The potential for long-distance transport of ARGs will be complemented by a 15-day low altitude transatlantic survey of airborne bioaerosols aboard a sailboat. Monitoring ARGs at sea constitutes a novel and innovative approach to better characterize ARG transport and identify the marine contribution to ARG emission worldwide. The campaign will be performed with the collaboration of the Blue Observer organization (www.blue-observer.com). It will consist of a 7000 km trip from Brest, France to Woods Hole, MA, USA, with daily air sampling using two different types of air collectors. A SASS3100 extractor will be mounted on the mast of a sailing ship (8–10 m high) and deployed daily for 1 h each night and each day. In parallel, three filter holders connected to individual pumps (7 L min^−1^) will be deployed continuously to perform 24 h sampling.

#### 3.2.3. Integrative Sampling Methods

In addition to using complementary methods and experimental designs, aspects of this project will incorporate and assess relatively novel methods that integrate air sampling with the realities and pressures introduced by anthropogenic changes to the environment. These integrative methods include the study the ARGs present in vehicle cabin air filters and on the conifer needle phyllosphere as proxies for long-term air sampling. Specifically, a modified version of the vehicle cabin filter method pioneered by Li and colleagues [33] and a new approach using the conifer needle phyllosphere as a biomonitor of airborne ARGs following Galés and colleagues [47]. These methods will allow for monitoring ARGs over longer temporal and geographic scales. Vehicle air filters have been collected from every province and territory of Canada and analyses are underway. To our knowledge, this method has only been performed once before [34]. However, it holds tremendous potential as the vehicle filters can construct an aggregate PM sample of a wide area at annual or biannual scales. It will be used to look for regional differences in airborne ARGs that could be linked to geographical or socioeconomic factors, such as landscape, land use, or population demographics.

The phyllosphere—referring to the cumulative aboveground plant biomass—is one of the largest biomes on Earth, 10^8^ km^2^ [48] supporting an estimated 10^7^ microbial cells cm^−2^ [49]. Phyllosphere microbial communities are strongly influenced by anthropogenic activities that emit microbes or alter deposition patterns [50,51]. Indeed, differences in ARG diversity of leaf-associated bacteria have been observed between agricultural and forest plants [51]. A pilot study on the efficacy of conifer needles as biomonitors of airborne ARGs has recently been published [52]. Briefly, conifer needles were collected near swine barns and in the farming community as well as the boreal forest to observe the diversity of ARGs in the phyllosphere from different environments. The needles were homogenized using a Stomacher (Aes Laboritoire, Bruz, France) and differentially centrifuged to generate pellets for DNA extraction. Differences were observed between the Boreal forest samples and those associated with human activities. This method holds great promise and will be expanded upon in upcoming experiments.

### 3.3. ARG Detection and Quantification

Determining the presence of ARGs in our diverse range of samples requires standardized methods. A shared ARG panel has been developed for qPCR analyses (Table 3). It is designed to capture a wide range of AMR. Since this type of nationwide project has not been previously undertaken in Canada, the panel was designed to be comparable to previous studies worldwide. The ARGs of interest were primarily selected from an array proposed by Stedfeldt and colleagues [53], but other genes were included at the suggestion of collaborators. For example, the colistin resistance gene *mcr-1* [54] was included due to its recent detection in swine feces in Québec [55]. A marker for the 16S rRNA gene will be used to provide biomass values and a reference point for ARG analyses [56]. We are employing a Takara SmartChip high-throughput qPCR system (TakaraBio USA, San Jose, CA, USA) to expedite sample processing using the shared ARG panel, in addition to validation using standard qPCR methods. For instance, bacterial biomass will be assessed via qPCR using the 16S rRNA marker gene.

### 3.4. Identification of Antimicrobial Resistant Bacteria

A selective culture approach with antibiotics will be used to target airborne antibiotic-resistant bacteria (ARB) carrying ARGs. Culture can retrieve less abundant bacteria which is one of the major biases of culture-independent methods [58]. This is the reason why air samples in the present work will be plated directly on solid agar media as well as inoculated in broths to enrich specific genera and/or specific types of ARB. Incubation in different atmospheres will allow the isolation of aerobic, micro-aerophilic, and anaerobic bacteria. All plates and broths will be supplemented with antibiotics, those of which will be chosen after known ARGs found in the type of environments studied, clinical isolates and for enrichment and inhibitory purposes. Isolated and purified colonies will be identified using MALDI-TOF mass spectrometry and DNA sequencing. Bacterial species will be further analyzed by antibiotic susceptibility testing against commonly used antibiotics, as previously described [59]. Whole-genome sequencing will permit species taxonomic assignation and detection of ARGs and their associated mobile genetic elements (MGEs) [60]. A shared DNA sequencing pipeline for metagenomics analyses will be developed and a suite of powerful tools for genome assembly, including Ray [61], Ray Meta for metagenome assembly [62], and Ray Surveyor for comparing metagenomes [63] will be deployed. Moreover, co-investigators have created machine-learning algorithms, such as KOVER [64], that can investigate important sequence features that can be associated with specific phenotypes including ARGs. Selective culture approaches will be performed on specific samples from hospitals, wastewater treatment plants, and livestock farms (Table 2).

Understanding the fate of ARGs and potential risks to human health is a key to this project. However, bacterial communities are shaped by a complex array of evolutionary, ecological, and environmental factors. As such, it is difficult to predict the fate of ARGs in environmental samples. The lack of functional demonstrations for ARGs in environmental metagenomes is a considerable limitation when characterizing the environmental resistome and assessing of its clinical relevance. Therefore, a selective culture approach will be employed to enrich bacterial species from selected samples collected at emission sources (i.e., hospitals, farms, wastewater treatment plants). The ARGs and or MGEs present in these isolates will be analyzed.

Furthermore, animal models will be developed to assess the risk of ARG transfer in vivo. Briefly, C57BL/6 mice will be treated with a mixture of antibiotics to perturb their gut microbiota over a period of five days. Liquid cultures of sorbitol peptone broth and bile salts media derived from air samples from selected environments will be introduced to the mouse gut once daily over the course of the eight-week experiment to mimic chronic exposure to potentially harmful bioaerosols. Faecal samples will be collected before, during, and after this process. DNA will be extracted from these samples and will be processed through the metagenomics pipeline described earlier.

### 3.5. Modeling

An atmospheric pollutant dispersion model will evaluate the dispersion and fate of bioaerosols and ARGs emitted from specific activities. Atmospheric dispersion models have been used extensively for both research and regulatory purposes. Previous research demonstrates the efficacy of dispersion models to estimate the risk of infection from bioaerosol exposure for residents near manure application sites [65]. Given the difficulty and expense of obtaining ambient air pollutant measurements over a large region, plume dispersion modeling is an effective alternative for assessing the impacts of bioaerosol sources on air quality in surrounding areas. The AERMOD [66] dispersion model will be used to determine the spatial distribution of bioaerosols and ARGs in proximity to their potential sources. AERMOD is approved for regulatory purposes by the U.S. Environmental Protection Agency and incorporates the Gaussian-plume modeling approach; a plume emanating from a source is considered to disperse in a manner analogous to the Gaussian distribution in both the y (lateral) and z (vertical) directions, whereas pollutants move by advection (wind transport) in the x (downwind) direction. AERMOD treats a pollutant as a non-reactive contaminant. It requires three fundamental inputs: the source type, the emission rate of the source, and local meteorological information to assess the emissions, and resulting downwind concentrations of bioaerosols from continuous emitters.

Maps of ARG and bacterial concentrations will also be produced to provide examples of plume development in a given direction to demonstrate how concentrations are reduced with distance away from sources. The same concentration maps will also be used to evaluate dispersion from short-term emissions, for example, from manure spreading. Results from this aspect of this research project will contribute to the development of information to inform policy decisions regarding safe distances from ARG sources to prevent their transmission to humans and domestic animals.

The roles of exposure pathways, bacterial relationships, co-selective pressures, ARG transmission, and the combined and cumulative effects of antimicrobial use need to be brought together in a One Health context. The One Health ethos posits that the interconnectedness of human, animal, and environmental activities are so great that they must be addressed together in an interdisciplinary manner to ensure health across systems and scales [67]. Integrated assessment models (IAM), developed for dealing with complex issues such as climate change, are a framework for organizing and processing evidence and uncertainties for complex systems in a complex manner, yet are still easily interpreted, ordered, and computationally efficient manner. An IAM specific to AMR in Canada (iAM.AMR) has been under development since 2015 [68]. The iAM.AMR quantitatively characterizes multiple linked transmission pathways of dissemination of AMR among humans, animals, and the environment. It uses a branching tree probability approach to modify the baseline probability of AMR using measures of association and the frequency of factor occurrence.

**Figure 2:**
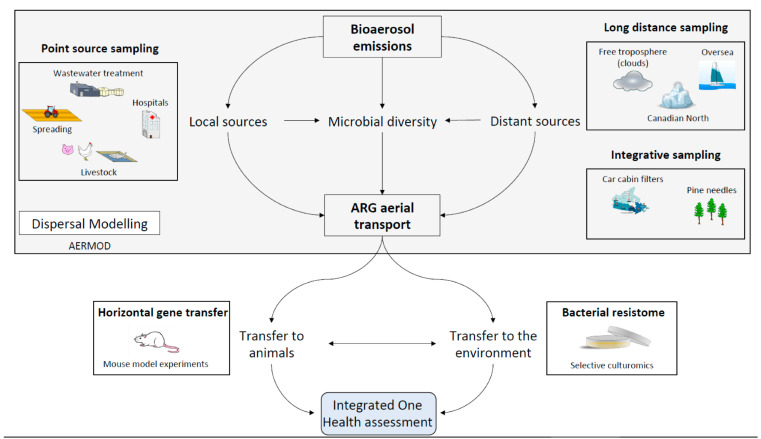
Conceptual diagram showing the creation of ARG risk assessment models. Data collected from the various study sites will be assembled to assess ARG emissions at point sources and over long distances. These will in turn inform ARG transfer studies in animal models and transport modeling. Together, these data will be used in an integrative ARG health risk assessment model. Ultimately this model aims to incorporate all relevant data to provide information to policymakers to make informed decisions to address the antibacterial resistance crisis.

To date, the iAM.AMR framework has been populated with a significant body of information on AMR and antimicrobial use from the scientific literature and surveillance data. Model development has focused on specific antimicrobial/bacteria/pathway combinations related to the food chain, particularly the poultry, swine, and beef production chains [68]. Our results will be added to the existing iAM.AMR framework to include AMR dissemination through bioaerosols in environmental pathways. This process will incorporate data from short and long-distance ARG transport models, as well as in vivo transfer in animal models. Ultimately, the aerosolized ARG transmission from multiple sources will assess the relative contributions of total transmission throughout the food chain, healthcare settings, watershed, and other systems, in order to identify optimal intervention points for AMR mitigation strategies (Figure 2).

### 3.6. Data Management

Given the interdisciplinary nature of this project, shared data collection and management practices must be implemented. The project will use a shared ARG panel (Table 3) and metagenomics pipeline. The limited selection of air samplers and standardized sampling design allows for uniform sampling techniques regardless of location, thus improving comparability of results from different locations and environments across Canada.

All researchers will upload their data to a secure, shared data portal. Data entry will conform to a standardized format to be accessible for all team members. This shared format will also permit the integration of different sampling campaigns into model design. These data can be selected or removed from models as needed by the lead statisticians without lengthy delays in contacting researchers for their data and reformatting them for specific uses.

## 4. Conclusions

Presently, the spread of ARGs in bioaerosols is not understood. This research program has the power to change paradigms by comprehensively addressing ARG emissions, exposure, and risk models, to inform policy for antimicrobial management and address AMR in the context of the environment. Antibiotic use and association with bioaerosols and geographical distribution of ARG across landscapes will be important knowledge produced from this work. Proof-of-concept for developing surveillance networks in natural and man-made environments using new rapid detection systems will be presented. Modeling will allow activity-based estimations of distribution. In addition, studying the contribution of ARG sources in bioaerosols will better contextualize our understanding of the contributions of activities to ARGs. The potential routes for transmission of antimicrobial-resistant bacteria and AMR genes between animals and people are complex and include many environmental routes beyond direct contact and the food chain. Thus, all data generated in this project will be made available to inform and expand an integrated exposure model on AMR.

The scientific data and models produced in this project will be accessible. This way, it is hoped our designs may be applicable to other countries and climates. The program addresses the complete ARG transport chain from sources, emissions, dispersal, and long-distance transport to models of exposure potential and risk in humans and animal models to elucidate the roles of bioaerosols in their transmission and detection of ARGs. These results have the potential for a far-reaching impact across Canada and internationally through collaborative team efforts, developing expertise and promoting the study of airborne ARGs to other anthropogenic and natural activities. Results will be important to policymakers, stakeholders, and communities to understand and develop strategies for mitigating ARG emissions. Ultimately, it is hoped that this project will serve as a blueprint for developing strategies to study bioaerosols and address AMR in the environment. 

## Figures and Tables

**Table 1:** Summary of sampling sites, number of samples, type of analysis, and outcomes.

Aims	Sampling Sites or Sample Type	Number of Samples	Analyses	Expected Outcome
**1**	Vehicle cabin filters	478 AC filters	qPCR total bacteria qPCR ARG panel DNA sequencing (subset)	Relative abundance of ARGs/bacteria Network analyses Mapping ARGs throughout Canada
**2**	Hospitals	100 air samples	DNA sequencing Culture	Network analyses Genomic and ARG profiles ARG enrichment-culturomics
	Wastewater treatment plants	100 air samples from beside aeration tanks (outdoor) or in the ventilation exit (indoor)	Meteorological data qPCR total bacteria qPCR ARG panel Culture DNA sequencing (subset)	Relative abundance of ARG/bacteria ARG transfer in animal model ARG enrichment-culturomics Network analyses
	Fish farm	24 indoor 24 outdoor 24 downwind 24 upwind	Meteorological data qPCR fish pathogen and mobile genetic elements in air, water, and sediments qPCR ARG panel	Relative abundance of ARG/bacteria Detection of mobile genetic elements
	Aquatic Containment Level 2 facility (LARSEM)	18	qPCR fish pathogen and mobile genetic elements	Transmission of ARGs in controlled setup
	Swine and poultry farms in depth analyses	2 swine barns 2 poultry barns (Quebec)	Meteorological data DNA sequencing qPCR ARG panel qPCR total bacteria Building ventilation properties	Relative abundance of ARG/bacteria Network analyses Emission rates Transport models ARG transfer in animal model ARG enrichment-culturomics Provincial and climatic variations
		2 swine barns 2 poultry barns (Saskatchewan)		
	Swine and poultry farms modest analyses	15 swine barns 8 poultry barns 1 poultry abattoir 1 swine abattoir (Quebec)	Meteorological data qPCR ARG panel	Seasonal variations Variation in emission rates of ARGs
		8 swine barns 8 poultry barns 1 poultry abattoir 1 swine abattoir (Saskatchewan)	qPCR total bacteria Building ventilation properties (estimation with CO_2_)	Transport models Province and climate variations
	Manure spreading	108 Swine slurries 36 Chicken manures with bedding 36 Chicken manures without bedding	Moisture content Meteorological data qPCR ARG panel DNA sequencing (subset) Geolocation and perception survey	Relative abundance of ARG/bacteria Network analyses Variation in emission rates of ARG Impact of spreading material and method Geolocation and perception model Transport models
**3**	In vitro ARG transfer study using samples from wastewater treatment plants and swine and poultry farms			
	Animal model of ARG transfer using samples from aims wastewater treatment plants and swine and poultry farms			
**4**	Conifer needles	Sampling gradient from known source Source sampling	qPCR total bacteria qPCR ARG panel	Proof of concept Transport model validation
**4**	Northern Canada	Ellesmere Island, Nunavut (50 samples) Resolute Bay, Nunavut (50 samples)	qPCR total bacteria qPCR ARG panel DNA sequencing (subset)	Long distance transport of ARGs Characterize Arctic resistome Transport model validation
**4**	Clouds	Puy-de-Dôme, France (15 samples)	qPCR total bacteria qPCR ARG panel DNA sequencing (subset)	Long distance transport of ARGs Describe remote spreading of ARGs Transport model validation
	Transatlantic	Transatlantic air samples (30 samples)		
	Precipitation	Opme meteo station (15 samples)		
**4**	Dispersion model using data from aims 2 and 3			
**5**	Integrated assessment model using data collected throughout the research program			

**Table 2:** Air extractors selected for aims 2 and 4.

Air Sampler	Type	Flow Rate (L/min)	Air Volume (m^3^)	Type of Analysis	Indoor/ Outdoor	Sites
SASS 3100	Electret filter	300	10	Molecular biology	I/O	Hospitals Wastewater treatment plants Fish farms Livestock buildings Manure spreading
SASS 4100	Electret filter + Virtual impactor	4000	100	Molecular biology	O	Northern Canada Fish farms
SASS 2300	Liquid cyclone	325	10	Molecular biology and culture	O	Hospitals Wastewater treatment plants Livestock buildings Manure spreading
Coriolis µ	Liquid cyclone	300	6	Molecular biology and culture	I/O	Hospitals Wastewater treatment plants Livestock buildings Manure spreading
High Flow Rate Impinger	Liquid impaction	530	100	Molecular biology and culture	O	Puy-de-Dôme, France

**Table 3:** List of shared gene targets and primers used for qPCR analyses. Genes noted by * used a FAM probe all others used SYBR Green fluorescence.

Gene	Gene Type	Primer Sequence	Ref.
16S rRNA *	rRNA gene—used here for biomass and reference	**F:** GGTAGTCYAYGCMSTAAACG **R:** GACARCCATGCASCACCTG **P:**TKCGCGTTGCDTCGAATTAAWCCAC-BHQ	[56]
*aac(6′)-II*	Aminoglycoside resistance	**F:** CGACCCGACTCCGAACAA **R:** CGACCCGACTCCGAACAA	[53]
*aac(6′)-Ib*	Aminoglycoside resistance	**F:** CGTCGCCGAGCAACTTG **R:** CGGTACCTTGCCTCTCAAACC	[53]
*aac(3)-iid_iii_iif_iia_iie*	Aminoglycoside resistance	**F:** CGATGGTCGCGGTTGGTC **R:** TCGGCGTAGTGCAATGCG	[53]
*blaCMY2*	Beta-lactam resistance	**F:** AAAGCCTCATGGGTGCATAAA **R:** ATAGCTTTTGTTTGCCAGCATCA	[53]
blaCTX-M-1,3,15 *	Beta-lactam resistance	**F:** CGTACCGAGCCGACGTTAA **R:** CAACCCAGGAAGCAGGCA **P:** CCARCGGGCZENGCAGYTGGTGAC	[57]
*blaGES*	Beta-lactam resistance	**F:** GCAATGTGCTCAACGTTCAAG **R:** GTGCCTGAGTCAATTCTTTCAAAG	[53]
*blaOXA*	Beta-lactam resistance	**F:** CGACCGAGTATGTACCTGCTTC **R:** TCAAGTCCAATACGACGAGCTA	[53]
*blaMOX/blaCMY*	Beta-lactam resistance	**F:** CTATGTCAATGTGCCGAAGCA **R:** GGCTTGTCCTCTTTCGAATAGC	[53]
*blaSHV-11*	Beta-lactam resistance	**F:** TTGACCGCTGGGAAACGG **R:** TCCGGTCTTATCGGCGATAAAC	[53]
*blaTEM*	Beta-lactam resistance	**F:** AGCATCTTACGGATGGCATGA **R:** TCCTCCGATCGTTGTCAGAAGT	[53]
*blaVEB*	Beta-lactam resistance	**F:** CCCGATGCAAAGCGTTATG **R:** GAAAGATTCCCTTTATCTATCTCAGACAA	[53]
*blaVIM*	Beta-lactam resistance	**F:** GCACTTCTCGCGGAGATTG **R:** CGACGGTGATGCGTACGTT	[53]
*erm(35)*	Macrolide resistance	**F:** CCTTCAGTCAGAACCGGCAA **R:** GCTGATTTGACAGTTGGTGGTG	[53]
*ermB*	Macrolide resistance	**F:** GAACACTAGGGTTGTTCTTGCA **R:** CTGGAACATCTGTGGTATGGC	[53]
*ermF*	Macrolide resistance	**F:** CAGCTTTGGTTGAACATTTACGAA **R:** AAATTCCTAAAATCACAACCGACAA	[53]
*ermT*	Macrolide resistance	**F:** GTTCACTAGCACTATTTTTAATGACAGAAGT **R:** GAAGGGTGTCTTTTTAATACAATTAACGA	[53]
*ermX*	Macrolide resistance	**F:** GCTCAGTGGTCCCCATGGT **R:** ATCCCCCCGTCAACGTT	[53]
*imp-marko*	Beta-lactam resistance	**F:** GGAATAGAGTGGCTTAATTC **R:** GGTTTAACAAAACAACCACC	[53]
*int1-a-marko*	Mobile genetic element	**F:** CGAAGTCGAGGCATTTCTGTC **R:** GCCTTCCAGAAAACCGAGGA	[53]
*is26*	Mobile genetic element	**F:** ATGGATGAAACCTACGTGAAGGTC **R:** CGGTACTTAATCTGTCGGTGTTCA	[53]
mcr-1 *	Colistin resistance	**F:** CACATCGACGGCGTATTCTG **R:** CAACGAGCATACCGACATCG	[54]
*qepA*	Quinolone resistance	**F:** GGGCATCGCGCTGTTC **R:** GCGCATCGGTGAAGCC **P:** CTACAGACCZENGACCAAGCCGA	[53]
*qnrB*	Quinolone resistance	**F:** TCACCACCCGCACCTG **R:** GGATATCTAAATCGCCCAGTTCC	[53]
*sul1*	Sulfonamide resistance	**F:** GCCGATGAGATCAGACGTATTG **R:** CGCATAGCGCTGGGTTTC	[53]
*sul2*	Sulfonamide resistance	**F:** TCATCTGCCAAACTCGTCGTTA **R:** GTCAAAGAACGCCGCAATGT	[53]
*tet32*	Tetracycline resistance	**F:** CCATTACTTCGGACAACGGTAGA **R:** CAATCTCTGTGAGGGCATTTAACA	[53]
*tetA*	Tetracycline resistance	**F:** CTCACCAGCCTGACCTCGAT **R:** CACGTTGTTATAGAAGCCGCATAG	[53]
*tetC*	Tetracycline resistance	**F:** ACTGGTAAGGTAAACGCCATTGTC **R:** ATGCATAAACCAGCCATTGAGTAAG	[53]
*tetL*	Tetracycline resistance	**F:** ATGGTTGTAGTTGCGCGCTATAT **R:** ATCGCTGGACCGACTCCTT	[53]
*tetM*	Tetracycline resistance	**F:**GGAGCGATTACAGAATTAGGAAGC **R:** TCCATATGTCCTGGCGTGTC	[53]
*tetO*	Tetracycline resistance	**F:** CAACATTAACGGAAAGTTTATTGTATACCA **R:** TTGACGCTCCAAATTCATTGTATC	[54]
*tetQ*	Tetracycline resistance	**F:** CGCCTCAGAAGTAAGTTCATACACTAAG **R:**TCGTTCATGCGGATATTATCAGAAT	[54]
*tetS*	Tetracycline resistance	**F:** TTAAGGACAAACTTTCTGACGACATC **R:** TGTCTCCCATTGTTCTGGTTCA	[54]
*tetW*	Tetracycline resistance	**F:** ATGAACATTCCCACCGTTATCTTT **R:** ATATCGGCGGAGAGCTTATCC	[54]
*tetX*	Tetracycline resistance	**F:** AAATTTGTTACCGACACGGAAGTT **R:** CATAGCTGAAAAAATCCAGGACAGTT	[54]
*tnpA*	Mobile genetic element	**F:** AATTGATGCGGACGGCTTAA **R:**TCACCAAACTGTTTATGGAGTCGTT	[54]
*vanA*	Vancomycin resistance	**F:** GGGCTGTGAGGTCGGTTG **R:** TTCAGTACAATGCGGCCGTTA	[54]
*vanB*	Vancomycin resistance	**F:** TTGTCGGCGAAGTGGATCA **R:** AGCCTTTTTCCGGCTCGTT	[54]
*vanRA*	Vancomycin resistance	**F:** CCCTTACTCCCACCGAGTTTT **R:** TTCGTCGCCCCATATCTCAT	[54]
*vanSA*	Vancomycin resistance	**F:** CGCGTCATGCTTTCAAAATTC **R:** TCCGCAGAAAGCTCAATTTGTT	[54]

Note: F indicates forward primer sequences; P indicates reverse primer sequences; P indicates FAM probe sequences.
